# Hydrogels from serum albumin in a molten globule‐like state

**DOI:** 10.1002/pro.3976

**Published:** 2020-10-22

**Authors:** Seyed Hamidreza Arabi, Behdad Aghelnejad, Jonas Volmer, Dariush Hinderberger

**Affiliations:** ^1^ Institut für Chemie Martin‐Luther‐Universität Halle‐Wittenberg Halle (Saale) Germany; ^2^ Département de chimie, École normale supérieure PSL University, Sorbonne Université Paris France

**Keywords:** albumin, ESR/EPR spectroscopy, fatty acids, hydrogels, hydrophobic effect, intermolecular interactions, ligand binding, molten globule, protein crowding, protein folding

## Abstract

We demonstrate that a molten globule‐like (MG) state of a protein, usually described as a compact yet non‐folded conformation that is only present in a narrow and delicate parameter range, is preserved in the high concentration environment of the protein hydrogel. We reveal mainly by means of electron paramagnetic resonance (EPR) spectroscopy that bovine serum albumin (BSA) retains the known basic MG state after a hydrogel has been formed from 20 wt% precursor solutions. At pH values of ~11.4, BSA hydrogels made from MG‐BSA remain stable for weeks, while gels formed at slightly different (~0.2) pH units above and below the MG‐state value dissolve into viscous solutions. On the contrary, when hydrophobic screening agents are added such as amphiphilic, EPR‐active stearic acid derivatives (16‐DOXYL‐stearic acid, 16‐DSA), the MG‐state based hydrogel is the least long‐lived, as the hydrophobic interaction of delicately exposed hydrophobic patches of BSA molecules is screened by the amphiphilic molecules. These bio‐ and polymer‐physically unexpected findings may lead to new bio‐compatible MG‐based hydrogels that display novel properties in comparison to conventional gels.

## 1 INTRODUCTION

Serum albumin is the most abundant protein in the circulatory system of a wide variety of organisms.^1^ In addition to being the major transport protein for un‐esterified fatty acids, it is also capable of binding reversibly an extraordinarily diverse range of metabolites, drugs and organic compounds and function as a carrier.^2^, ^3^, ^4^ This protein was and still is studied extensively concerning its structure and functionalities. Among others, electron paramagnetic resonance (EPR) spectroscopy has developed into a powerful analytical tool.^5^, ^6^

Developing new biocompatible materials which have an affinity towards therapeutic substances and can carry them inside a living organism is an attractive concept in the biomedical sciences. Hydrogels based on proteins or synthetic molecules are good candidates for this task since one of their main common features is biocompatibility. Their high water content and soft nature make them similar to natural extracellular matrices and minimize tissue irritation and cell adherence.^7^ In addition, their capacity to embed biologically active agents in their water‐swollen network makes them very attractive for controlled release of therapeutics.^8^ In a more general perspective, hydrogels are cross‐linked networks made out of hydrophilic polymers, capable of retaining large amounts of water but yet remaining insoluble and maintaining their three‐dimensional structures. The reason behind this insolubility is that the polymer is physically and/or chemically cross‐linked.^9^, ^10^, ^11^, ^12^ Albumin hydrogels have previously been formed by thermal denaturation and chemical cross‐linking.^13^, ^14^, ^15^, ^16^, ^17^ These hydrogel systems typically require either extensive protein denaturation (thermal) or chemical modification, which can hamper protein functionality and compromise biocompatibility. Another method called electrostatically triggered albumin self‐assembly introduced by Baler et al.^18^, ^19^ takes advantage of the fact that albumin has the ability to reversibly change its conformation when exposed to changes in solution pH while at least partly preserving secondary structure elements that may act as functional binding domains. We recently demonstrated that albumin hydrogels can in fact be formed well below their denaturation temperature if enough time is granted and explored the large pH range in which the hydrogels are formed. We also introduced supporting phase diagrams that precisely allow implementing hydrogels with specific properties on the nano‐ and macroscopic scales.^19^ A globular protein varies its tertiary structure under different solvent conditions. Native and fully denatured states are the most prominent ones. There is another important state that is thought to be the common intermediate in protein folding which is called molten globule (MG). Even four decades after its proposed discovery, the MG state of proteins still remains enigmatic.

There is substantial debate on its nature, ranging from MG states either reflecting a general intermediate in protein folding to being unique for every protein or simply a low‐energy misfolded species. In this work, further complexity is added to the current discussion around MG states by demonstrating that molten globule‐like state of protein, BSA, can be preserved in hydrogels, formed by the protein itself. In the context of our paper, one may define that molten globules are partially folded conformations of proteins that have near native compactness, substantial secondary structure, slowly fluctuating (little detectable) tertiary structure and an increased solvent‐exposed hydrophobic surface area relative to the native state.^20^

## 2 RESULTS AND DISCUSSION

In the following, the presence of the molten globule‐like states both in acidic and basic pH ranges of bovine serum albumin (BSA) are investigated in high concentration environments with the help of continuous‐wave electron paramagnetic resonance (CW EPR) spectroscopy. We first develop the spectroscopic analysis for a 5 wt% albumin solution (below the gel point) and then, for the first time, describe the same experiments performed with BSA hydrogels at high concentrations (20 wt%). We present evidence that the MG state observed in individual proteins persists in the gel state of the protein and in fact dominates its properties.

In case of human serum albumin (HSA), there is a molten globule state, which can be obtained under acidic conditions at pH 2.0.^21^, ^22^ This state was recognized with the help of different techniques including far‐UV and near‐UV circular dichroism (CD), binding of (8‐anilinonaphthalene‐1‐sulfonic acid) ANS as a hydrophobic probe and viscosity measurements. In addition, Reichenwallner et al.^23^ confirmed the presence of the known MG state at pH 1.90 ± 0.20 by CW EPR spectroscopy on EPR‐active stearic acid derivatives that bind to the protein. The first MG state of BSA that is well established was detected in alkaline BSA solutions at pH 11.2 and confirmed several times.^24^, ^25^, ^26^ Like in the mentioned case of HSA, the conclusion that BSA is present in an MG state was reached by analyzing far‐UV and near‐UV CD spectra. There are some experimental results that acknowledge the second potential MG state of BSA in the acidic pH range. One study has focused on the combined effects of concentration and pH on the conformational states of BSA by the means of small‐angle X‐ray scattering and came to the conclusion that there is in fact an acidic molten globule like state (pH 2.0).^27^ Another independent research study came to a largely similar conclusion about the existence of the acidic MG like state for BSA. The method of choice was extrinsic and intrinsic protein fluorescence and it was reported that BSA assumes this conformation at pH 3.0.^28^

**FIGURE 1:**
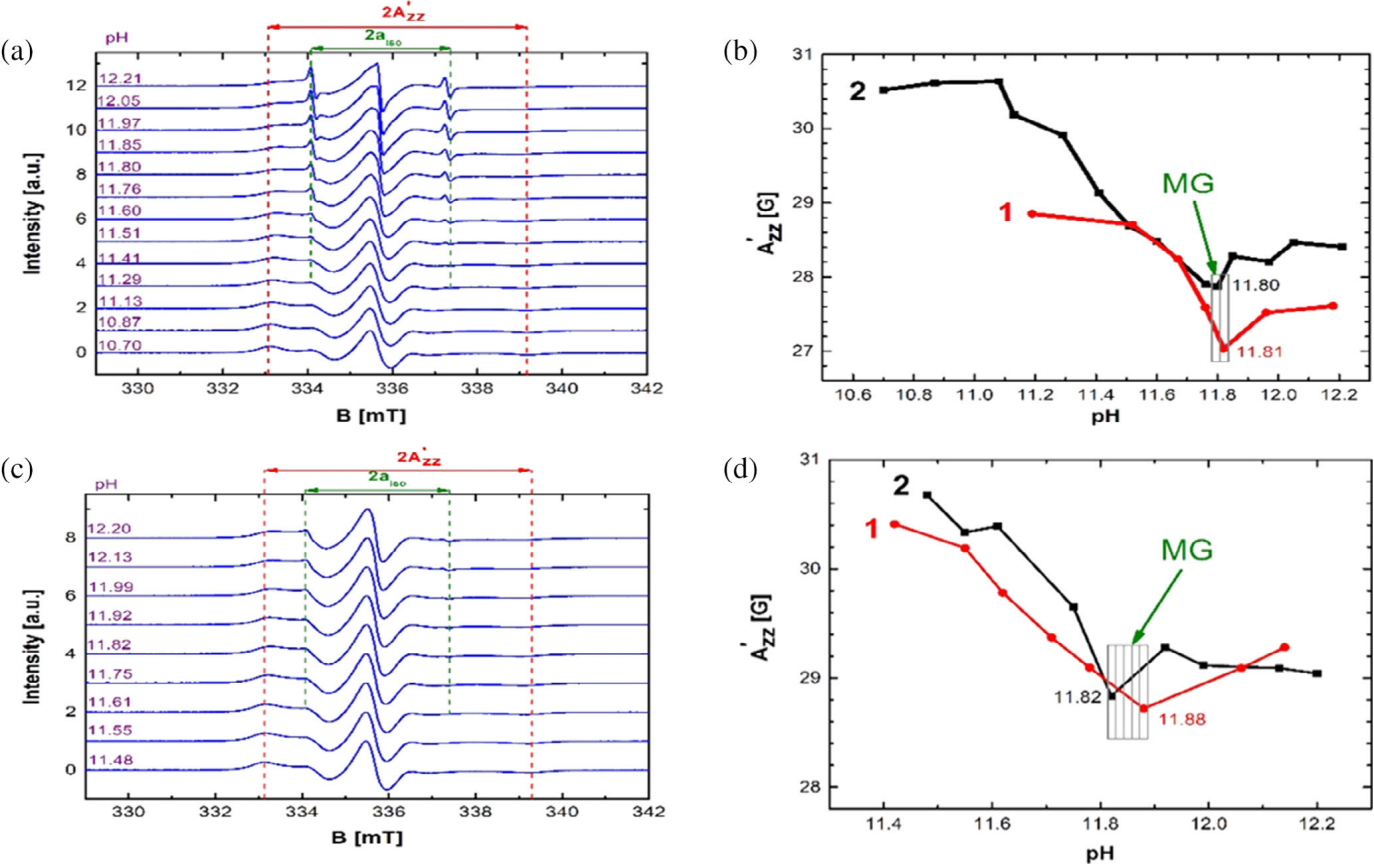
(a) EPR spectra of 16‐DSA in diluted 5 wt% BSA solutions in the basic pH range at 20°C (1:1 ratio of BSA:16‐DSA). (b) Dependence of $A_{\mathrm{zz}}^{\prime }$ on pH in diluted BSA solutions in the basic pH range at 20°C (two independent measurement series). (c) EPR spectra of 16‐DSA in 20 wt% BSA hydrogels in the basic pH range at 20°C (1:1 ratio of BSA:16‐DSA). (d) Dependence of $A_{\mathrm{zz}}^{\prime }$ on pH in BSA hydrogels in the basic pH range at 20°C (two independent measurement series). Please note the difference in axis scaling between (b) and (d)

Figure 1 displays the results in the basic pH range. Figure 1a presents a pH‐dependent series of 16‐DSA spectra in diluted 5 wt% albumin solutions. The isotropic hyperfine coupling constant (*a*_iso_) peaks of freely tumbling 16‐DSA appear from pH 11.29 onwards and the intensity of these peaks increases with increasing pH, while the broad spectrum of albumin bound 16‐DSA (indicated by the apparent hyperfine coupling constant $A_{\mathrm{zz}}^{\prime }$ in the spectra) decreases in intensity. As explained in detail in Reference 23, this is due to the protein changing into the so‐called aged form of albumin, in which particularly the tertiary structure and parts of the secondary structure elements are largely dissolved, reducing the number of binding regions in the protein and leading to release of 16‐DSA.^23^

On the other hand, the value of apparent hyperfine coupling constant $A_{\mathrm{zz}}^{\prime }$ is a spectroscopic determinant of the polarity of the molecular environment.^29^ Polar environments lead to larger hyperfine splittings and reversibly, the hyperfine splittings are smaller in apolar surroundings.^29^ One of the characteristics of the molten globule state is an increased solvent‐exposed hydrophobic surface area. There is a higher accessibility to hydrophobic parts of the protein in this state. In this case, fatty acids can easily access and bind to these exposed areas. Now, because those spin probes reside longer times in apolar surroundings, the value of $A_{\mathrm{zz}}^{\prime }$ decreases. Therefore, the point in Figure 1b in which $A_{\mathrm{zz}}^{\prime }$ is at its minimum can be associated with the molten globule state.^23^ Another aspect relating the minimum of $A_{\mathrm{zz}}^{\prime }$ to the MG state is that the minimum of $A_{\mathrm{zz}}^{\prime }$ also denotes the maximum rotational mobility of the spin probes. This stems from the fact that for fast and isotropic rotations of nitroxide spin probes, the apparent spectral separation of the outermost extrema ($A_{\mathrm{zz}}^{\prime }$) converges towards its minimum value and approach the typical three‐line pattern. Hence, when rotational mobility increases at the MG state, these spectral features move closer together, as it can be seen in Figure 1, It is widely accepted that the molten globule is a compact state with some fluctuating side chains. Here, the fatty acids interact with the exposed hydrophobic patches in the MG state, while conventionally their binding sites are rigid channels in the protein's interior, which gives them increased mobility. Two independent series of experiments were performed and reported in Figure 1b (first series is marked in red and the second one in black). It can be seen that both of them have a minimum at approximately pH 11.8. When considering that there is an uncertainty in the pH measurements (± 0.05) and apparent hyperfine coupling constant readouts, the fact that two completely independent series of tests show two minima close to each other (within less than 0.1 pH units) reaffirms the notion that there is a molten globule like state at this point. The previously reported pH value for the basic BSA molten globule state is 11.2^24^, ^25^, ^26^ which is reasonably close to this research's finding. In another research work that used the CW EPR method, but with different buffer and protein concentrations, 11.8 was reported as the pH value, where the protein is in a molten globule like state.^23^ For a more detailed quantitative description of the different 16‐DSA binding and rotational motion modes, the reader is referred to Reference 23. The discrepancy in pH of the MG state (at pH 11.2 and 11.8 in EPR spectroscopy on 16‐DSA) can be assigned to the presence of 16‐DSA. We and others have previously shown that the presence of fatty acids stabilizes the native structure of albumin^19^ and thus seems to shift the molten globule state to slightly but significantly higher pH.

Figure 1c,d depicts the same pH‐dependent series of 16‐DSA EPR spectra, this time in BSA hydrogels made from 20 wt% precursor solutions. Tumbling of the spin probes is more restricted in hydrogels due to a denser protein environment than in solution. Unlike before, the freely rotating species are emerging at a higher pH of approximately 11.60 (instead of 11.30). Similar to the diluted experiments, there appears to be a drop in $A_{\mathrm{zz}}^{\prime }$, with a broad minimum centered at around pH 11.80 ± 0.2 and a noticeable increase after that. It should be noted, though, that the drop in the apparent hyperfine coupling when increasing pH is much shallower than that observed for the dilute BSA solutions ($\Delta A_{\mathrm{zz}}^{\prime }$ ~ 2.0 G in gels vs. ~3.0 G in dilute solution). These results suggest there could be a molten globule like state in the hydrogels. This further suggests that the functionality of the protein is mostly preserved during the electrostatically triggered method of the BSA gelation and even though the binding, configurations and even the macroscopic phase of the whole system is changed, the delicate state of the molten globule survived the process (see Figure S1 to compare the behavior of 16‐DSA in presence and absence of protein in basic solution).

The same series of experiments have been conducted on the MG states acidic ranges (see Figure S2). In essence, we could not find experimental evidence that the acidic MG state can persist in BSA hydrogels. Hence, we here further focus on and elucidate the basic MG‐state in BSA hydrogels.

**FIGURE 2:**
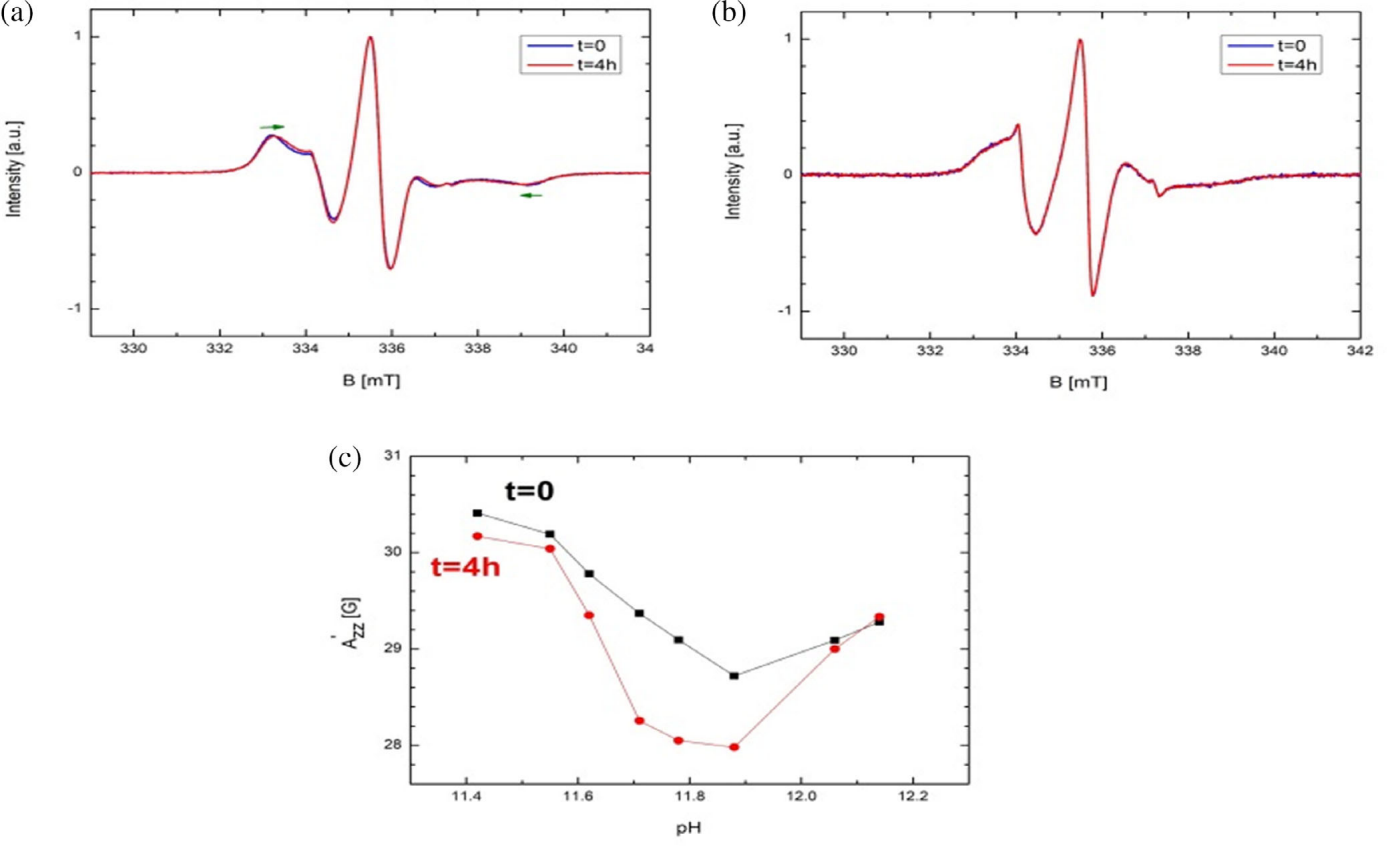
(a) EPR spectra of 16‐DSA in 20 wt% BSA hydrogel with pH 11.55 at 20°C (1:1 ratio of BSA:16‐DSA). (b) EPR spectra of 16‐DSA in 20 wt% BSA hydrogel with pH 11.5 at 37°C (1:1 ratio of BSA:16‐DSA). (c) Dependence of $A_{\mathrm{zz}}^{\prime }$ on the pH at different times in 20 wt% BSA hydrogels in the basic pH range at 20°C

As we know from our earlier studies,^19^ the effect of time on (basic and other) hydrogels is an important issue worth discussing. Therefore, one series of experiments was conducted in two stages. The first stage was performing EPR spectroscopy on the 16‐DSA containing samples after the gelation started (denoted *t* = 0, in fact about 10 min after start of the gelation) and the second stage was the repetition of the same measurements after approximately 4 hr (*t* = 4 hr). The difference between these spectra revealed an unexpected characteristic (Figure 2c). It can be seen that after 4 hours the spectrum displayed a prominent change and $A_{\mathrm{zz}}^{\prime }$ was decreased, that is, the outer extrema in the spectra moved closer to each other. This could be due to the fact that although at *t* = 0 the sample was, macroscopically seen, completely in the gel state, but the gelation process apparently was not completely finished and some sort of curing progressed slowly at the molecular level. This example was measured at pH 11.55, but all samples at different pH values showed these changes (Figure 2a). All the previous EPR measurements were performed at 20°C. Switching to 37°C reveals the effect of temperature on the gelation process (Figure 2b). After 4 hr the spectrum is identical to that measured at *t* = 0. This again proves the notion that temperature expedites the gelation process. Taking into account the time‐dependent curing, the minimum apparent hyperfine coupling $A_{\mathrm{zz}}^{\prime }$ in the pH‐dependence can now clearly be ascribed to the pH region 11.80 ± 0.2 and the $\Delta A_{\mathrm{zz}}^{\prime }$ also increases to ~2.5 *G*.

The CW EPR spectra strongly suggest that during and after the gelation process the albumin molecules persist in their MG state so that 16‐DSA molecules can attach to the exposed hydrophobic areas of the BSA‐MG. This finding is remarkable, as in a 20 wt% protein solution that consequently gelates, the system seems dominated by intermolecular contacts and extensive overlap between different albumin molecules. Yet, the presumably delicate MG state that energetically may be seen either as intermediate folding state or being a local energy (enthalpic and entropic) minimal state in these rather harsh conditions (pH > 11), seems robust enough to even persist the regime of strong intermolecular interaction posed by the gel state.

To further substantiate this remarkable EPR‐derived conclusion, we expanded our research portfolio. As stated above, in literature there are different ways to verify the MG state in proteins. However, most of them are not applicable in this research work. IR spectroscopy cannot be used, as the pH range needed (above 10) is higher than the allowed pH limit of ATR‐IR spectrometers' crystals. Moreover, viscosity measurements and rheological characterizations in general cannot be employed. Here, we are working with very high protein concentration and pH ranges. Accordingly, the gelation is a matter of seconds even at low temperatures (e.g., room temperature). Therefore, with these standard rheological methods it is almost impossible to get meaningful data of the initial stages of the gelation process. Furthermore, the data collected for low concentration media (Figure S4) show no substantial pH‐dependent difference between the samples, making it unlikely to detect differences during the gelation process.

We aimed at exploring transmission electron microscopy (TEM) on lower concentration samples at pH 11.8 but the images are not conclusive and do not give additional information (Figure S3).

**FIGURE 3:**
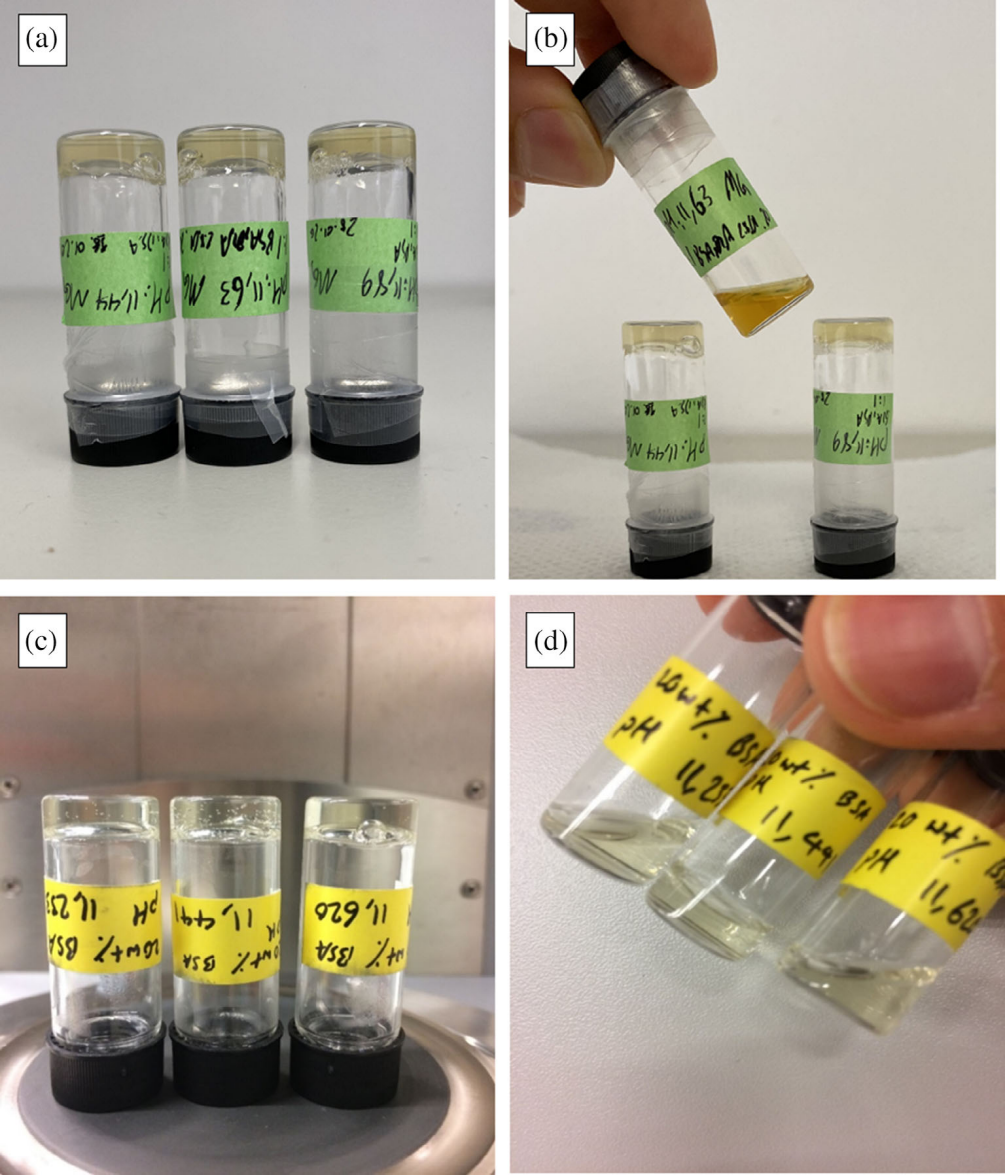
(a) 20 wt% BSA hydrogels with fatty acids (16‐DSA) at ~pH 11.233, 11.441, 11.626 at 20°C (all the samples in gel state). (b) The same samples after 3 weeks (21 days) kept at RT. Only the sample with the pH value 11.65 turned into a viscous solution and lost its hydrogel 3D structure and water holding capacity, while at the other two pH values the gels remain intact. (c) 20 wt% BSA hydrogels without fatty acids at ~pH 11.25, 11.45, 11.65 at 20°C (all the samples in gel state). (d) The same samples after 3 weeks (21 days) kept at RT. Except for the sample with the pH value 11.45 the other turn into viscous solution and lost their hydrogel 3D structure and water holding capacity

It may sound trivial, but one may also emphasize the importance of simple visual inspection of samples. In this case, we observed that the gelation process for all the samples (above a certain albumin concentration and pH value, based on previously reported phase diagram of albumin gelation, see Reference 19) took place in a few minutes. Astonishingly, when leaving the hydrogels (with or without 16‐DSA) at RT for several weeks, we observed a seemingly paradoxical behavior of the gels. On the one hand, the hydrogels made in the presence of 16‐DSA remained in gel state after at least 3 weeks with one remarkable exception, namely at pH ~11.65. In other words, the gels made at pH values only slightly (~0.2) above or below this value (between pH 11 and 12.2) all remain in a gel state (BSA:16‐DSA ratio of 1:1) but the hydrogel made at pH 11.65 which initially and for up to 3 weeks was in the gel state turned into a viscous solution. Figure 3a,b illustrate this phenomenon. In Figure 3b the color change is noticeable. As Hall et al. showed the turbidity of protein solution increases as the aggregate size increases.^30^ This indicates that there is protein aggregation at pH 11.65 in the solution and the 3D structure lost its water holding capacity and as a result it is not a gel anymore but only a cloudy solution, giving a darker, colloidal‐like impression. On the other hand, all the hydrogels made without 16‐DSA at all pH values above 11 turn into viscous solutions after several (at least two) weeks, with the exception of the hydrogel made at pH value ≃11.4 (without 16‐DSA, Figure 3c,d, Table S1, and Video S1). Considering the known stabilization against denaturation of BSA due to presence of long‐chain fatty acids like 16‐DSA (e.g., Reference 19, 23) and comparing the values to the measured minimum of $A_{\mathrm{zz}}^{\prime }$ in EPR spectroscopy, we can conclude that BSA molecules in the gel with 16‐DSA at pH ~11.65 and in the gel without 16‐DSA at pH ~11.4 are in the MG state. This would mean that the MG state does not only survive the gel‐formation process and the gel state but it also seems to dominate the properties of the gel compared to non‐MG state gels.

The clear deterioration of albumin without FAs in the non‐MG state at high pH values, leads us to the conclusion, that the gelation process is likely kinetically controlled and that at the high pH values per se it is a rather thermodynamically unfavorable process. Only in the MG state the deterioration of the gelation network does not proceed.

**FIGURE 4:**
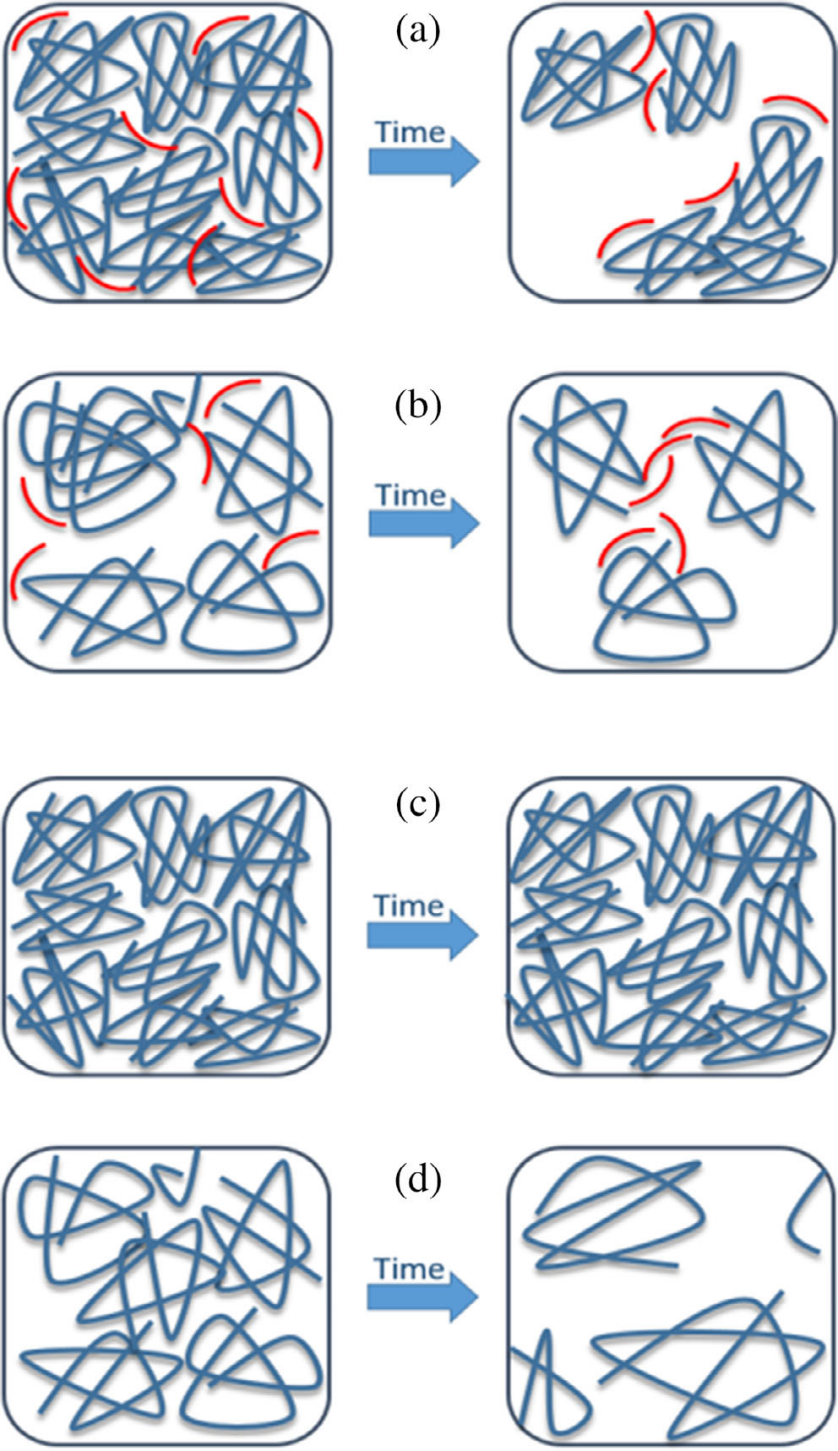
(a) Schematic suggested structure of the gels in compact MG state. 16‐DSA (shown as red chains, the drawing is not to scale, for the sake of illustration 16‐DSA molecules are drawn bigger compared to protein chains shown in blue) screening of the hydrophobic interactions between MG‐BSA leads to destabilization of the gels in the MG‐state. (b) Suggested structure of the gels, made at pH values where the MG state does not appear. In this state, due to presence of 16‐DSA, the hydrogels show very high stability for several weeks. 16‐DSA may act acts as secondary structure stabilizer and bridge BSA molecules to keep the 3D structure stable. (c) Suggested structure of the gels in the compact MG state without fatty acids. These gels remain in the stable gel‐state for several weeks (thermodynamically stable) as BSA molecules can interact through their hydrophobic patches, which are presented on the outside in the MG state. (d) Suggested structure of the gels without fatty acids, made at pH values where the MG state does not appear. In this case although gels form initially (kinetically controlled), after several weeks the 3D structure of the gels is destroyed and its water holding capacity is compromised, such that a viscous solution ensues (thermodynamically controlled)

One may interpret this on the molecular level as schematically shown in Figure 4. Serum albumin in the MG state has been shown to expose more hydrophobic areas to the solvent compared to its native state, where most of the hydrophobic areas are hidden inside the heart shaped protein. This may explain why gels made at the pH value where individual albumin molecules occupy the MG state persist in the gel state for weeks, while those at pH values even only slightly higher or lower than the MG pH at ~11.4 deteriorate with time (Figure 4c,d). Apparently, the compact MG state (one deals with a known basic contraction in albumins in the aged form above pH ~11) forms gels that are structurally stable.

However, when 16‐DSA is introduced in the hydrogels, these FAs screen the hydrophobic intermolecular interactions between BSA molecules that is essential for keeping the three‐dimensional scaffold of the gels.

In this case, the more exposed hydrophobic patches of BSA in the MG state can readily be targeted as loose attachment sites by amphiphilic 16‐DSA molecules, which constitutes more favorable thermodynamic (entropy driven) state. Figure 4a shows that presence of 16‐DSA might thus screen the hydrophobic interactions between individual BSA molecules in the MG state gels and destabilize the gel overall. This might explain the color change observable in Figure 3b as protein aggregates in a cloudy, nanocolloidal solution could lead to the observed darkening.

One may further conclude that when BSA is not in the MG state, residual classical binding sites for FAs are again available ($A_{\mathrm{zz}}^{\prime }$~ is larger again, indicating stronger immobilization) which may again stabilize secondary structure elements and such, indirectly, the gel. Amphiphilic FAs may also form hydrophobic bridges from the less exposed hydrophobic areas between BSA molecules and consequently stabilize these gels as shown in Figure 4b.

The MG state of the individual proteins is a thermodynamically stable (or metastable intermediate) conformational state.^31^, ^32^ Serum albumin in the MG state has been shown to expose more hydrophobic areas to the solvent compared to its native state, where most of the hydrophobic areas are hidden inside the heart shaped protein. This may explain the contradictive behavior of the MG state gels. On the one hand, these exposed hydrophobic areas stabilize the gel for several weeks in contrast to other gels made with only slightly different pH values. On the other hand, they can destabilize the gel when the hydrophobic interaction is hampered and screened due to the presence of hydrophobic fatty acid chains.

As depicted schematically in Figure 4c, the increased long‐term stability of MG‐state hydrogels may thus on the molecular level be due to increased exposition of hydrophobic patches (as we see it in EPR spectroscopy; Figure 1).

Being compacted, the intermolecular network formation between individual albumins can take place despite the large negative charge that the molecules have at this pH (net charge around −80, see Reference 23). The hydrophobic contacts persist while the large amounts of water and counterions entropically trapped in the gel lead to partial screening of the surface charges. This gel state is stable for weeks, similar to gels formed, for example, by thermal denaturation at native pH values.^33^ Table S1 in the SM shows the concentration dependence of this process. As schematically illustrated in Figure 4d, at the pH‐points above or below the MG state, the individual albumin molecules cannot form a stable gel that can persist longer than a few days. This indicates that thermodynamically the entropic trapping of water inside a protein network does not persist and only initially a kinetically trapped gel forms. The gel may break down because the molecules change their conformational states (they are not in the stable MG state), lose their relative compactness and cannot form the network through hydrophobic interactions. Instead, the large negative charge becomes relatively more important and like‐charge repulsion leads to looser individual conformations and overall self‐assembly.

This peculiar phenomenon cannot be seen in hydrogels synthesized in acidic pH ranges. Apart from the fact that gel formation in acidic ranges takes much longer (several hours compared to a few minutes in basic ranges), these hydrogels are water soluble before the leaching process^19^ and after the leaching, despite their enhanced mechanical properties.

## 3 CONCLUSIONS

In conclusion, the existence of basic molten globule states for BSA hydrogels (and the non‐existence in the acidic range hydrogels) has been shown with the help of CW EPR. This method takes advantage of the fact that there are more exposed hydrophobic patches in the MG state in comparison to the native or unfolded states that can then be detected by CW EPR spectroscopy on amphiphilic fatty acid derivatives (16‐DSA). The MG state hydrogel without this fatty acid (pH 11.4) is the maximum stability point for the gel state (Figures 3 and S4) that can even be loaded with and manipulated by co‐dissolved small molecules. Contrarily, the MG state hydrogel with fatty acid features the shortest stability time (at pH 11.6) and the hydrogels turn into viscous solution while the hydrogels made with slightly lower or higher pH values remain in hydrogels stated for several more weeks. The slight difference between these two values (11.4 and 11.6) can be traced back to the presence of 16‐DSA in one of them which slightly shifts the pH in which MG state gel forms. These results are robustly confirmed with EPR spectroscopy.

To the best of our knowledge, the presence of the molten globule‐state in the high‐crowding environment of a hydrogel is proposed in the present research for the first time. Furthermore, we made the observation (in independent measurements by different experimenters) of enhanced gel stability at the special pH value of the MG‐state. These results indicate a new path for experimental studies of MG states in proteins (and their analysis from a “polymer under constraints” point of view) and given the broad spectrum of dynamic protein structures, reaching from globally folded via MG to intrinsically disordered states it may be of interest to test the persistence of many MG state proteins at high crowding conditions. By addition of equimolar amounts of other amphiphilic molecules like long‐chain fatty acids, one may even apparently fine‐tune of intermolecular interactions and thus the macroscopic gel properties. Even though the pH values needed for BSA MG hydrogels are too high to be of direct practical use, one may envisage new biocompatible materials (probably from proteins in MG states that are induced at less harsh conditions) with unique properties which can have practical use in biomaterials science such as in drug delivery applications and beyond. From a fundamental protein biophysical point of view, it is remarkable that the enigmatic molten globule state can apparently be very robust and not only persist in the high concentration environment of a hydrogel but even dominate the gels nanoscopic and macroscopic properties.

## 4 MATERIALS AND METHODS

Our experimental strategy is based on co‐dissolution of BSA and hydrophobic 16‐DOXYL‐stearic acid spin probes (also used before, e.g., in References 19, 23) to achieve a ratio of 1:1 c/c of 16‐DSA and BSA. For each experiment, a certain amount of acid (2 M HCl stock solution) or base (2 M NaOH stock solution) was added to a previously prepared solution until the pH set point was reached (Orion Versa Star Pro pH Benchtop Meter). The gelation time was varied from 5 to 45 min in the basic pH range. For gelation in the acidic pH range, more time and higher temperature are required. Therefore, in this case they were first put in the capillaries and then the capillaries were incubated in a water bath at 37°C for 20 hr. The prepared capillaries were placed in the resonator cavity of a Magnettech Miniscope MS 400 (Magnettech Berlin, now part of Bruker) benchtop EPR spectrometer (X‐band range) and after reaching the desired temperature, the spectra were recorded.

The parameter used for EPR measurements included microwave frequency of approximately *ν* = 9.4 GHz and a magnetic field sweep of 15 mT centered at 340 mT. Temperatures were adjusted with a Temperature Controller H03 (Magnettech) with an accuracy of ∼0.2°C. The exact frequency was recorded using a frequency counter (Racal Dana 2101, Neu‐Isenburg, Germany).

## AUTHOR CONTRIBUTIONS

**S. Arabi:** Data curation; investigation; methodology; visualization; writing‐original draft; writing‐review and editing. **Behdad Aghelnejad:** Data curation; formal analysis; investigation; validation; visualization; writing‐original draft; writing‐review and editing. **Jonas Volmer:** Data curation; formal analysis; investigation; validation; writing‐original draft. **Dariush Hinderberger:** Conceptualization; formal analysis; funding acquisition; investigation; methodology; project administration; resources; supervision; visualization; writing‐original draft; writing‐review and editing.

## Supporting information


**Data S1** Additional EPR experiments at high and low pH values, TEM micrograph and viscosity measurements.

**Video S1** Short video showing the robustness of the MG‐state hydrogel.
