# Supplementary material for: Hydrogels from serum albumin in a molten globule‐like state
Source: Protein Sci. 2020 Oct 22;29(12):2459–67. doi: 10.1002/pro.3976 (PMC7679958; doi:10.1002/pro.3976)
Supplement: Supplementary file 1 — Data S1 Additional EPR experiments at high and low pH values, TEM micrograph and viscosity measurements. [file PRO-29-2459-s001.docx]

***Supplementary Material***

**Hydrogels from serum albumin in a molten globule-like state**

Seyed Hamidreza Arabi †^1^, Behdad Aghelnejad †^2^, Jonas Volmer ^1^ and Dariush Hinderberger*^1^

1. Institut für Chemie, Martin-Luther-Universität Halle-Wittenberg, Von-Danckelmann-Platz 4, Halle (Saale), 06120, Germany.

2. Département de chimie, École normale supérieure, PSL University, Sorbonne Université, CNRS, 75005 Paris, France.

***Corresponding author**: Institut für Chemie, Martin-Luther-Universität Halle-Wittenberg, Von-Danckelmann-Platz 4, Halle (Saale), 06120, Germany, phone: +49-345-55-25230; fax: +49-345-55-27576; [www.epr.uni-halle.de](http://www.epr.uni-halle.de); E-Mail: [dariush.hinderberger@chemie.uni-halle.de](mailto:dariush.hinderberger@chemie.uni-halle.de)

† These authors contributed equally.


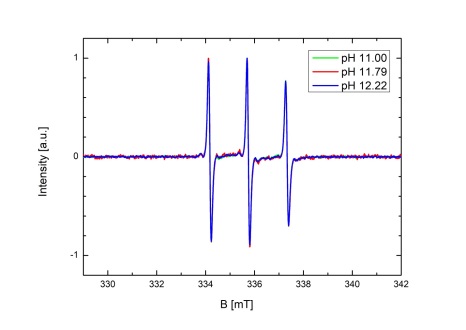
EPR spectra of 16-DSA alone in the basic pH range at 20 °C.

**Figure S1**. 16-DSA in basic solution at different pH values. The pH values were set using 2 M NaOH. There is no remarkable difference in the behavior of 16-DSA at different pH values in absence of protein.

Figure S2 explores the possibility of existence of a molten globule like state for BSA in the acidic pH range. As the case before, EPR spectra of 16-DSA in the albumin hydrogel in a wide pH range were measured and apparent hyper-fine couplings were analyzed (Figures S1 A, B). There is no sharp drop in the apparent hyperfine coupling constant. But still the fact that two completely unrelated series of experiments show coinciding minima at nearly the same point supports the idea that there probably is a molten globule like state at approximately pH 2.2 for diluted 5 wt. % BSA precursor solutions. Comparing with the literature, even though it is not as established as the MG state in the basic pH range, there have been some indications pointing to a MG like state at pH 2 ^1^ and pH 3 ^2^. In addition, another research work (by implementing the same EPR technique) came to the conclusion that pH 2.4 is the point at which the molten globule state is reached ^3^. An interesting observation that is mentioned by Barbosa et al. ^1^ is that they detected their proposed MG state for BSA in the acidic pH range for 25 and 50 mg/ml concentrations (2.5 and 5 wt. %), but they did not detect it with a concentration of 10 mg/ml (1 wt. %) implementing the same method. Their proposition is that the emergence of this state is in fact concentration dependent.

Additional experiments on the acidic gel state of BSA showed that all the spectra are similar to each other at the acidic pH ranges (Figure S1 C). There are two possible explanations for these results. Firstly, the samples were prepared the day before the measurements and they were incubated in a water bath at 37 °C for 20 hours. The time and the temperature could have an aging effect on the gels and the delicate molten globule state cannot survive them. The second factor that could play a role is the protein concentration; because these gels were synthesized by 20 wt. % BSA precursor solutions and as it was explained earlier, concentration can play a role on weather an MG state is formed or not. It is obvious that there is no MG state present in acidic BSA hydrogels.


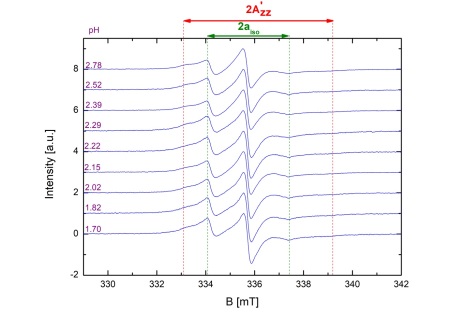

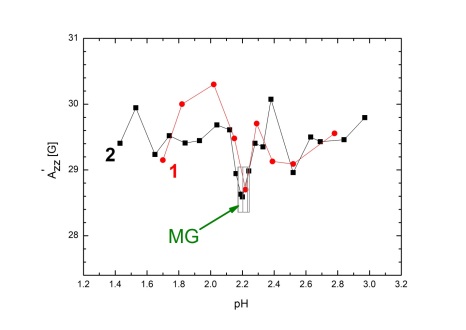

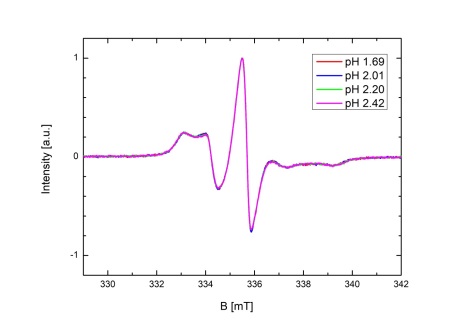

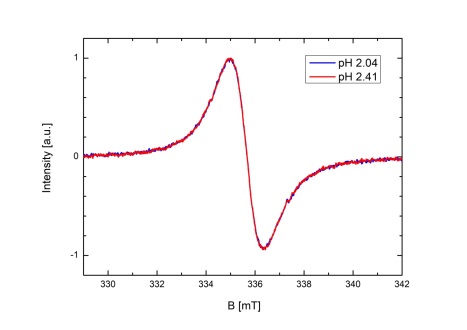


A)

B)

C)

D)

**Figure S2.** (A) EPR spectra of 16-DSA in diluted 5 wt. % BSA solutions in the acidic pH range at 20 °C (1:1 ratio of BSA:16-DSA). (B) Dependence of A’zz on the pH in diluted 5 wt. % BSA solutions in the acidic pH range at 20 °C (2 independent experiment series). (C) EPR spectra of 20 wt. % BSA hydrogels in the acidic pH range at 20 °C. (D) EPR spectra of 16-DSA in the acidic pH range at 20 °C.

Reference spectra without protein show one broad (spin-exchange dominated) line, which allows the conclusion that the system almost completely consists of aggregated and micellar fatty acids with just a small number of freely tumbling ones (Figure S2 D).

Figure S3 demonstrates a recorded TEM image for a sample with pH 11.77. Even though 1 wt. % protein solution was used for this TEM experiment, the local concentration must have increased to a much higher value (close to the gel concentration), when it is dried on the grid. Therefore, these images can give us an insight about hydrogels themselves.

In the image the black arrow points to a group of aligned protein fibers. Even though the contrast is not very distinct, these protein fibers seem to be parallel to each other. On the other hand, the white arrow points a bundle of fibers which are probably on top of each other. Therefore, there seems to be a better and clearer contrast in this area. Finally, odds are that the area which the blue arrow points at, is just accumulated staining agent and that does not represent a morphological feature of our sample. Even though TEM experiments were performed on samples with different pH values, the images were not helpful for detecting the MG state. This fact could be due to a number of reasons. First of all, the samples were exposed to a staining agent with pH 8. So inevitably the resulting pH would have been an unknown and different value in the end. Secondly, it takes some time for the sample to be completely dried on the grid. The MG state, as delicate as it is, most probably cannot survive that condition. In addition to that, there is no solvent interaction in the sample, because it is dry now. Thirdly, the resolution is not good enough to see what is happening to the protein molecules in detail. As it was shown earlier, only protein fibers were observed.


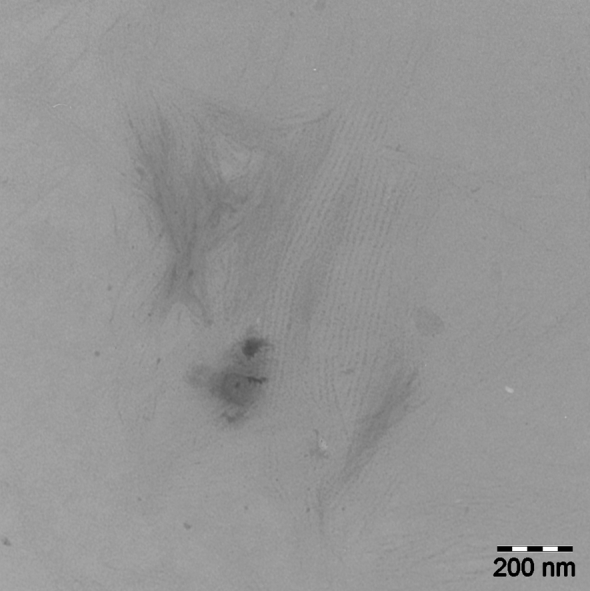


**Figure S3.** TEM micrograph obtained from 1 wt. % BSA solution at pH 11.77

Even in low concentration samples the viscosity measurements show no apparent difference among sample with different pH values. Figure S4 A shows the behavior of 5wt% (0.75 mM) serum albumin at T0 (beginning of the process). The measurements were done from the same vials of samples after 21 days. Figure S2 B shows that even at low protein concentration the sample with pH around 11.4 differentiate itself from other samples.


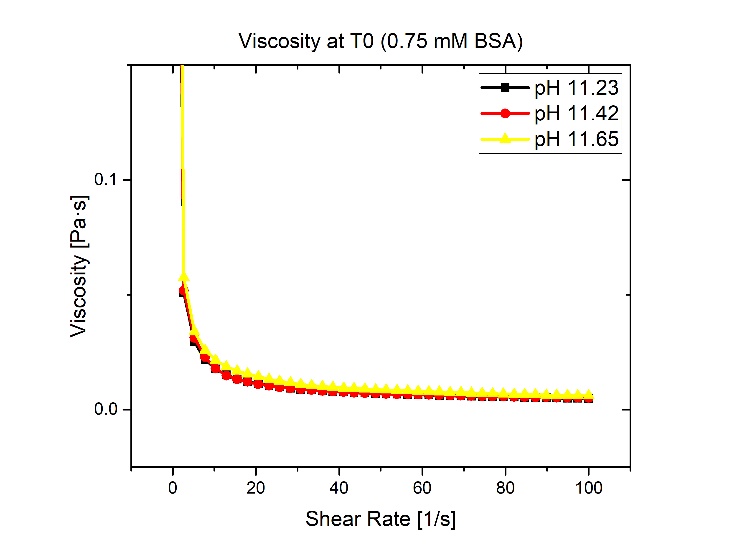

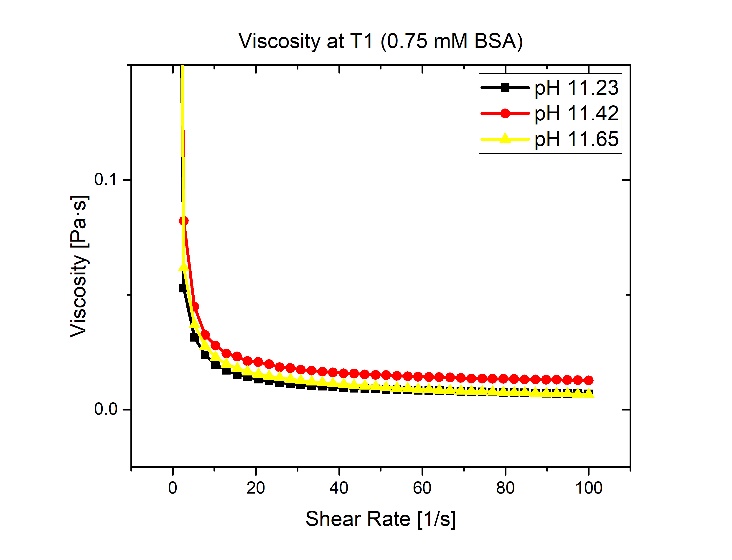


**Figure S4.** (A) Viscosity measurements at 20⁰C from 0.75 mM BSA stock solution, with pH values set to 11.2, 11.4 and 11.6 using 2M NaOH. (B) for the same samples (kept at RT for three weeks (21 days)) the viscosity measurements were done at 20⁰C. Here we see that the sample with pH 11.4 shows higher viscosity. T0 is the day of measurements and T1 shows the same measurements using same batches of samples which were kept for 21 days at room temperature.

(A)

(B)

Table S1 shows how the behavior of hydrogels changes with time from 20 wt% (3mM) BSA stock solutions. The pH values were set to the show values using 2M NaOH.

**Table S1.** State of hydrogels as a function of time.

| Sample Nr. | pH value | Week 1 | Week 2 | Week 3 | Week 4 |
| --- | --- | --- | --- | --- | --- |
| 1 | 11.003 | Gel/Sol | Viscous solution | Fluid | fluid |
| 2 | 11.226 | Gel | Gel | Viscos solution | fluid |
| 3 | 11.438 | Gel | Gel | Gel | Gel |
| 4 | 11.618 | Gel | Gel | Viscos solution | Viscos solution |
| 5 | 11.850 | Gel | Gel | Viscos solution | Viscos solution |

Figure S5: Precursor solutions (all 20 wt. % BSA (3mM)) are loaded with 16-DSA. 16-DSA is dissolved in 0.1M KOH prior to addition into precursor solution. Based on the results without the presence of 16-DSA (see Fig. 4) samples at pH above 11.2 all turned into hydrogels at room temperature within one hour. However, addition of 16-DSA hindered samples with pH below 11.6 from gel formation (at least for one hour). This explains the pH difference in EPR results (the minimum of apparent hyperfine coupling around pH 11.8) and the pH at which hydrogels stay in gel state after several weeks (despite gels at pH 11.2 and 11.6, which both lose their gel structure and water holding capacity).


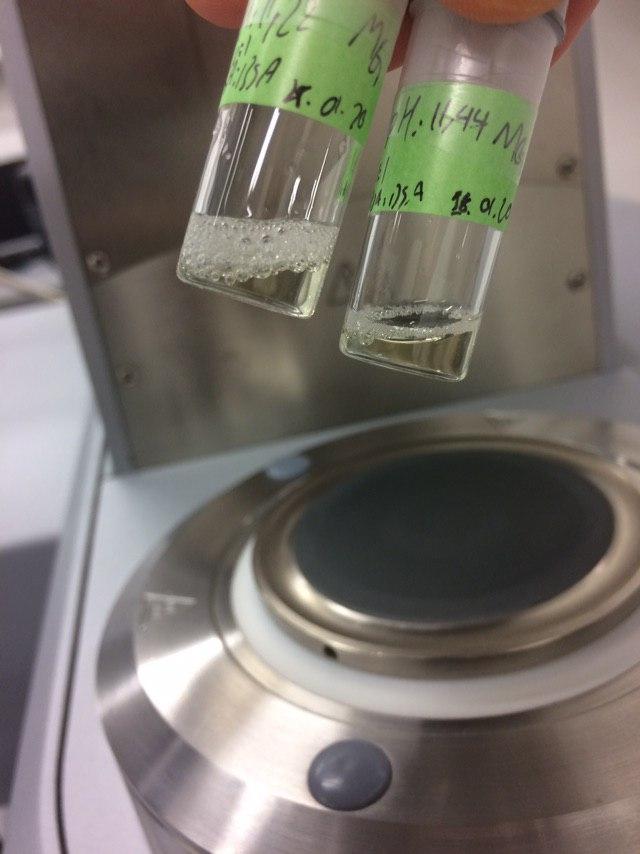

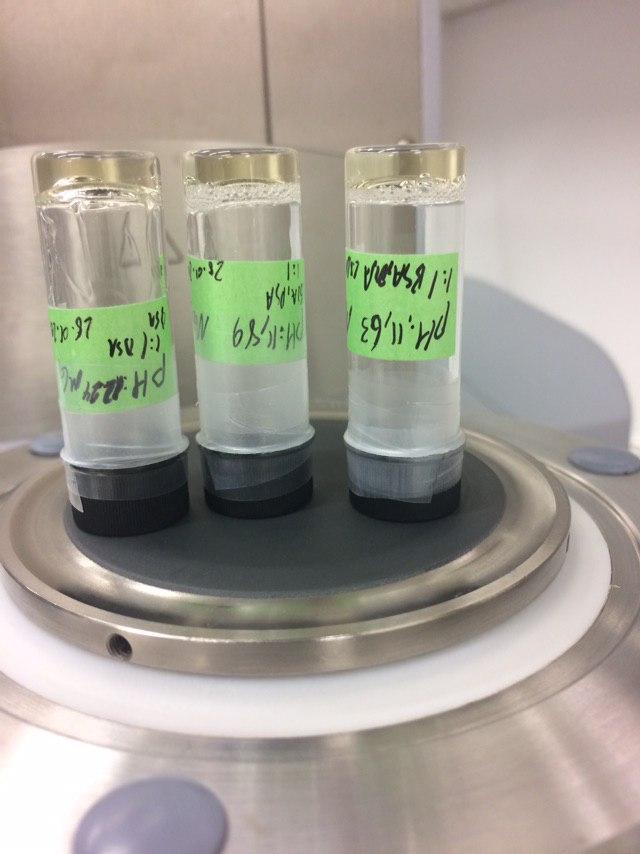


A

B

**Figure S5.** (A) 20 wt. % BSA precursor solution with 16-DSA (1:1 BSA:FA ratio) at pH 11.22, 11.44 at 20 °C did not turn into gel after one hour (B) 20 wt. % BSA precursor solution with 16-DSA (1:1 BSA:FA ratio) at pH 11.63, 11.89, 12.24 at 20 °C, samples turned into gel in less than one hour.

V1. Video Link (please put the video link here):

1. L. R. Barbosa, M. G. Ortore, F. Spinozzi, P. Mariani, S. Bernstorff and R. Itri, *Biophysical journal*, 2010, **98**, 147-157.

2. M. Bhattacharya, N. Jain, K. Bhasne, V. Kumari and S. Mukhopadhyay, *Journal of fluorescence*, 2011, **21**, 1083-1090.

3. J. Reichenwallner, M.-T. Oehmichen, C. E. H. Schmelzer, T. Hauenschild, A. Kerth and D. Hinderberger, *Magnetochemistry*, 2018, **4**, 47.
